# Is patient-centredness in European hospitals related to existing quality improvement strategies? Analysis of a cross-sectional survey (MARQuIS study)

**DOI:** 10.1136/qshc.2008.029397

**Published:** 2009-01-26

**Authors:** O Groene, M J M H Lombarts, N Klazinga, J Alonso, A Thompson, R Suñol

**Affiliations:** 1 Avedis Donabedian Institute, Autonomous University of Barcelona, and CIBER Epidemiology and Public Health (CIBERESP), Barcelona, Spain; 2 Academic Medical Center, Department of Social Medicine, University of Amsterdam, Amsterdam, the Netherlands; 3 School of Social and Political Studies, University of Edinburgh, Scotland

## Abstract

**Background:**

There is growing recognition of patients’ contributions to setting objectives for their own care, improving health outcomes and evaluating care.

**Objective:**

To quantify the extent to which European hospitals have implemented strategies to promote a patient-centred approach, and to assess whether these strategies are associated with hospital characteristics and the development of the hospital’s quality improvement system.

**Design:**

Cross-sectional survey of 351 European hospital managers and professionals.

**Main outcome measures:**

Patients’ rights, patient information and empowerment, patient involvement in quality management, learning from patients, and patient hotel services at the hospital and ward level were assessed. The hypothesis that the implementation of strategies to improve patient-centredness is associated with hospital characteristics, including maturity of the hospital’s quality management system, was tested using binary logistic regression.

**Results:**

In general, hospitals reported high implementation of policies for patients’ rights (85.5%) and informed consent (93%), whereas strategies to involve patients (71%) and learn from their experience (66%) were less frequently implemented. For 13 out of 18 hospital strategies, institutions with a more developed quality improvement system consistently reported better results (percentage differences within maturity classification ranged from 12.4% to 46.6%). The strength of association between implementation of patient-centredness strategies and the quality improvement system, however, seemed lower at the ward than at the hospital level. Some associations (OR 2.1 to 5.1) disappeared or were weaker after adjustment for potential confounding variables (OR 2.2 to 3.7).

**Conclusions:**

Although quality improvement systems seem to be effective with regard to the implementation of selected patient-centredness strategies, they seem to be insufficient to ensure widespread implementation of patient-centredness throughout the organisation.

There is growing recognition in the literature of the contribution patients can make to setting objectives for their own care,^1^ ^2^ improving health outcomes,^3^ ^4^ or evaluating healthcare.^5^ Given patients’ and the public’s expectations, the nature of chronic disease management, and the potential economic benefits of involving citizens and patients in their care, there is relatively broad agreement on the need for a patient-centred approach in health service delivery.^6^ ^7^

The concept of patient-centredness has a long tradition in medicine and health services research, going back to the 1950s. At that time, the excessive focus of medical care on disease processes rather than the illness experience raised many concerns and initiated calls for a more holistic biopsychosocial model of health.^8^ ^9^ A universally accepted definition of patient-centredness, however, does not exist.^10^ For example, the Institute of Medicine defines it as^7^:

“health care that establishes a partnership among practitioners, patients, and their families (when appropriate) to ensure that decisions respect patients’ wants, needs, and preferences, and that patients have the education and support they need to make decisions and participate in their own care.”

Stewart,^11^ in her definition, emphasises exploring the patients’ main concerns, emotional, and information needs, finding common ground, and enhancing the continuing relationship between patient and doctor. The Picker approach to assessing patients’ experience defines eight dimensions of patient-centred care^12^:

- access;
- respect for patients’ values, preferences and expressed needs;
- coordination and integration of inpatient services;
- information, communication, and education; physical comfort;
- emotional support and alleviation of fear and anxiety;
- involvement of family and friends;
- transition and continuity after discharge.

Based on a study of primary care delivery, Little *et al* summarised patient-centredness as follows^13^:

“There are at least three important and distinct domains of patient-centredness: communication, partnership, and health promotion.”

Although a patient-centred approach is widely advocated, its implementation in practice is limited and related to characteristics of both doctors and patients.^14^^–^16 There is evidence that patients frequently do not receive important information on their condition and options for self-management, and that there is insufficient involvement of patients in developing quality goals.^17^ ^18^ Moreover, surveys frequently report patients’ dissatisfaction with the way services are organised in the hospital,^19^ the lack of time for consultations, and difficulties in understanding what doctors tell them.^20^ These shortcomings of hospital services on measures of patient-centredness may be explained partly by the lack of systematic evaluation and integration of activities into the hospital’s quality management system: although hospital quality management systems usually address generic issues through policies on patients’ rights and informed choice, and conduct surveys of patients’ views, patients’ involvement in decisions concerning their disease, or in the design of new services, seems not yet to have become a focus of hospital quality management systems.^21^

In order to promote patient-centredness, baseline information is required on the implementation of related strategies and on existing implementation gaps. Moreover, clarification is needed whether the domains of patient-centredness should be promoted as an integral part of existing quality management systems, or through the development of new, separate systems. We therefore examined the implementation of strategies applied in European hospitals to improve patient-centredness, and their relationship with existing quality management systems. Our specific objectives were to:

- quantify the extent to which European hospitals have implemented strategies to promote a patient-centred approach
- determine whether the adoption of these strategies is associated with hospital characteristics and the maturity of the hospital’s quality improvement system.

## MATERIAL AND METHODS

### Setting

Our study was based on a cross-sectional survey of hospitals in Belgium, the Czech Republic, France, Ireland, the Netherlands, Poland, Spain, and the UK. Acute care hospitals with more than 100 beds were identified by country coordinators, and hospitals were randomly selected from this group of centres. Selected hospitals were contacted to participate in the study and to complete an electronic questionnaire. The sampling characteristics and hospital recruitment criteria used in this study are explained fully elsewhere.^22^

### Data collection

The questionnaire included four sections: section 1 (strategic level, sent to the chief executive officer or quality manager), which included a general description of the hospital’s structures and quality management systems, and sections 2–4 (ward level, sent to the head of the department), which included quality measures related to the management of acute myocardial infarction (AMI), appendicitis and deliveries (details about the questionnaire design are described elsewhere in this supplement^22^). Data on deliveries will be presented in a separate paper.

The questionnaire design was based on a review of quality improvement questionnaires, literature reviews, analysis of accreditation manuals and additional studies carried out within the Methods of Assessing Response to Quality Improvement Strategies (MARQuIS) project.^23^^–^25 Data collection took place between April and August 2006. The questionnaire was translated into five languages (Czech, Dutch, French, Polish, Spanish) based on a protocol for forward and backward translation.

### Operationalisation of patient-centredness

We assessed patient-centredness at two levels: the hospital management level and the ward level. Patient-centredness at the hospital level was operationalised according to the following main domains, identified through a literature search: patients’ rights; patient information and empowerment; patient involvement in quality management; learning from patients; and patient hotel services. For each domain we included items that were evaluated as most relevant according to the conceptualisation in the literature. We excluded the domain “patient involvement in quality management” from operationalisation at the ward level. Because our study did not involve data collection at the level of individual patients, we excluded domains such as reduction in anxiety and emotional support from our operationalisation of patient-centredness.

### Quality improvement maturity

In order to test associations between patient-centredness (dependent variable) and hospital characteristics and the development of the hospital’s quality management system (independent variables), we classified hospital quality improvement maturity in this study. This classification integrates seven dimensions reflecting overall developmental phases (maturity) of the hospitals’ quality improvement system: policy, planning and documents (20 items), leadership (36 items), structure (19 items), general quality improvement activities (8 items), specific quality improvement activities (20 items), patient involvement in quality management (6 items) and accountability (4 items). This classification does not include ward-level data. The process of developing and validating the maturity classification and its scoring and cut-off points are described in detail elsewhere in this supplement.^26^

### Statistical analysis

For this study we excluded the patient-centredness items included in the original maturity classification to avoid relating the same items, thus reducing the number of items from 113 to 95. Cronbach’s α (before: 0.75, after 0.78), mean and standard deviation changed only slightly. We observed only minor changes in the distribution of hospitals in quartiles (κ  = 0.826, p<0.001).

For dependent variables (strategies to improve patient-centredness at the hospital level and the ward level) we used the Picker approach to create a problem score, based on the four original response categories.^12^ As independent variables we considered hospital characteristics, grouping on the quality improvement maturity classification, country and ward level. Two countries contributed data for only a few hospitals (the Netherlands: n = 10, the UK: n = 14) so we excluded them from the analysis. We used univariate and bivariate statistics to describe the extent to which European hospitals had implemented strategies to improve patient-centredness, and assessed associations with independent variables using the χ^2^ test (Pearson χ^2^ and linear-by-linear association for assessment of rank). We then used binary logistic regression (stepwise, entry of variables at 0.05, removal at 0.1) to test the hypothesis that the implementation of strategies to improve patient-centredness is associated with hospital characteristics such as maturity of the hospital’s quality management system, ownership, type, ward, and country effects.

## RESULTS

Of the 389 hospitals that participated in the MARQuIS project, 351 contributed usable data for our classification of quality improvement maturity. Table 1 provides an overview of the distribution of hospitals by criteria such as ownership, type, size, and maturity group.

**Table 1:** Hospital characteristics by maturity classification

Characteristics	Total(N = 351)n (%)	High maturity(N = 87)n (%)	Intermediate maturity(N = 177)n (%)	Low maturity(N = 87)n (%)	p Value*
Ownership					0.006
Public	271 (80.9)	61 (76.3)	130 (76.9)	80 (93.0)	
Private not-for-profit	34 (10.1)	8 (10.0)	24 (14.2)	2 (2.3)	
Private for-profit	30 (9.0)	11 (13.8)	15 (8.9)	4 (4.7)	
Total	335 (100)	80 (100)	169 (100)	86 (100)	
Hospital type					0.364
University hospital	78 (23.6)	18 (23.1)	42 (25.3)	18 (20.9)	
General, residency training	163 (49.4)	31 (39.7)	85 (51.2)	47 (54.7)	
General, non-teaching	89 (27.0)	29 (37.2)	39 (23.5)	21 (24.4)	
Total	330 (100)	78 (100)	166 (100)	86 (100)	
Hospital beds					0.091
<200	60 (18.8)	18 (23.4)	26 (16.1)	16 (19.5)	
200–399	97 (30.3)	23 (29.9)	53 (32.9)	21 (25.6)	
400–599	63 (19.7)	13 (16.9)	33 (20.5)	17 (20.7)	
600–799	38 (11.9)	14 (18.2)	19 (11.8)	5 (6.1)	
800–999	22 (6.9)	2 (2.6)	13 (8.1)	7 (8.5)	
>999	40 (12.5)	7 (9.1)	17 (10.6)	16 (19.5)	
Total	320 (100)	77 (100)	161 (100)	82 (100)	

*Pearson χ^2^ test (linear-by-linear association for assessment of rank).

Of the 351 hospitals included, the majority (271, 80.9%) were under public ownership. We observed significant differences in the distribution of hospital ownership by maturity classification (p = 0.006). Private for-profit hospitals were more strongly represented in the group with a high maturity classification (13.8%), whereas private not-for-profit hospitals were almost absent in the group with a low maturity classification (2.3%); however, the absolute numbers were very low. The distribution of hospital type was more homogeneous, but did differ significantly; about a half of the hospitals (49.4%) were characterised as general hospitals with residency training, and about one quarter each either university (23.6%) or non-teaching hospitals (27%). The number of hospital beds ranged widely from <200 to >999 beds. In our study, hospital size (number of beds) was not clearly related to the maturity classification.

Table 2 summarises the data on the implementation of strategies to improve patient-centredness at the hospital level according to the maturity classification. In general, hospitals in the high maturity group reported better implementation of strategies to improve patient-centredness, and we observed significant differences for 13 of the 18 items. Considering items related to patients’ rights, we observed an overall high self-reported implementation rate for posting patients’ rights (85.5%) and written policies for confidentiality (85%), with significant differences by maturity grouping only for the latter. Almost all hospitals reported that in general, consultation and treatment rooms allowed privacy. With regard to patient information and empowerment, we observed that informed consent policies appeared to be widely implemented; however, policies for patient involvement and responsibilities for health promotion appeared to be much less common. Hospitals in the more mature groups were significantly more likely to have implemented such strategies.

**Table 2:** Hospital-wide implementation of strategies to improve patient-centredness by maturity classification

Domain Item	Total (%)n = 351	High maturity (%)(n = 87)	Intermediate maturity (%)(n = 177)	Low maturity (%)(n = 87)	p Value*
Patients’ rights					
Patients’ rights posted	85.5	85.9	87.4	81.4	0.403
Consultation and treatment rooms allow privacy	97.1	100.0	96.5	95.3	0.067
Written policy on confidentiality	85.0	96.5	86.5	70.6	<0.001
Patient information and empowerment					
Written policies for informed consent	93.0	95.3	95.3	85.9	0.016
Written policies for patient involvement	70.6	89.4	69.4	53.7	<0.001
Responsible person for health promotion	59.9	86.1	57.3	39.5	<0.001
Patient involvement in quality management					
Involved in development of criteria or standards	21.4	38.5	18.4	11.1	<0.001
Involved in design of protocols	19.3	28.9	18.2	12.3	0.009
Involved in evaluation of quality objectives	35.1	51.3	30.3	29.1	0.004
Involved in participation in quality committee	30.1	33.8	24.2	38.5	0.533
Involved in participation in improvement project	39.6	50.6	32.3	43.6	0.361
Involved in discussion of results of patient survey	35.4	44.9	30.6	35.8	0.242
Learning from patients					
Internal quality improvement includes monitoring patients views	65.5	89.5	64.1	43.4	<0.001
Internal quality improvement includes analysis of patients’ complaints	86.3	96.6	88.4	71.8	<0.001
Data on complaints reported to governing board	91.7	96.4	94.7	81.0	<0.001
Data on monitoring patients’ opinion reported to governing board	82.9	93.8	84.5	68.4	<0.001
Patient hotel services					
Possibility of obtaining a single room upon request	63.2	81.9	59.4	53.2	<0.001
Choice in timing of meals	11.5	17.5	11.6	5.1	0.015

*Pearson χ^2^ (linear-by-linear association for assessment of rank).

Patient involvement in quality management was not a common strategy among the hospitals participating in our study, and appeared to be associated with the maturity classification only for selected items. We detected clear differences between maturity classification groups in the extent to which hospitals reported “learning from patients”: hospitals in the most mature group were much more likely to report learning from patients than hospitals in the remaining groups. One exception was handling patients’ complaints, which in general were reported to the hospitals’ governing board irrespective of maturity classification. Finally, with regard to patient hotel services we observed that single beds on request and choice in timing of meals were significantly associated with maturity classification; it is worth noting though that in general only 11.5% of all hospitals offered a choice in the timing of meals.

Table 3 summarises the cross-tabulations of the implementation of patient-centredness strategies at the ward level by maturity classification. We report the results of χ^2^ tests with correction for rank for differences between maturity classification, and χ^2^ tests for differences between wards.

**Table 3:** Ward-specific implementation of patient-centredness strategies by maturity classification

Domain Item	Ward	Maturity classification (%)			p Value for difference in maturity classification*
		High	Intermediate	Low	
Patients' rights					
Patients give written consent to treatment	AMI	68.7	67.5	53.1	0.059
	Appendicitis	81.7	72.5	66.7	0.114
	p Value ward difference†	0.076	0.334	0.081	
Consultation and treatment allow privacy	AMI	73.9	66.9	63.4	0.378
	Appendicitis	74.0	75.2	67.1	0.410
	p Value ward difference	0.994	0.108	0.625	
Patient information and empowerment					
Ability to inform foreign patients about their condition and treatment	AMI	50.7	42.5	34.7	0.394
	Appendicitis	45.8	40.4	25.7	0.119
	p Value ward difference	0.811	0.354	0.784	
Patients informed at discharge about follow-up care	AMI	89.0	94.3	93.8	0.326
	Appendicitis	94.7	94.7	89.9	0.325
	p Value ward difference	0.210	0.867	0.360	
Learning from patients					
Patients invited to express opinion	AMI	36.8	33.8	33.3	0.874
	Appendicitis	44.0	38.3	36.3	0.586
	p Value ward difference	0.370	0.403	0.698	
Patients informed about complaints procedure	AMI	80.0	66.0	59.8	0.021
	Appendicitis	89.5	71.7	50.0	<0.001
	p Value ward difference	0.105	0.280	0.212	
Patient hotel services					
Single room on request	AMI	65.2	45.2	42.7	0.011
	Appendicitis	62.0	47.3	44.3	0.063
	p Value ward difference	0.699	0.711	0.836	
Choice in timing of meals	AMI	11.6	10.2	2.4	0.070
	Appendicitis	16.4	8.4	3.8	0.025
	p Value ward difference	0.407	0.596	0.609	

*χ^2^ test with linear-by-linear association for assessment of rank between maturity classification.

†χ^2^ test for differences between wards.

AMI, acute myocardial infarction.

These analyses confirmed the associations between strategies to improve patient-centredness and the revised maturity classification grouping at the hospital level (table 2). In other words, hospitals in the most mature group consistently reported better implementation of patient-centredness strategies, even though this trend was not always statistically significant. Nevertheless, the association appeared to be weaker at the ward level than at hospital level. For example, although almost all hospitals reported hospital-wide strategies to ensure privacy, the statement that “consultation and treatment allow privacy” was much less frequently answered in the affirmative at the ward level than at the hospital level—even in the most mature groups (73.9% for AMI, and 74.0% for appendicitis). Similar differences in reporting at the hospital and ward level were observed for the other items. Implementation of strategies to learn from patients was low. In the most mature group only 36.8% (for AMI) and 44.0% (for appendicitis) of wards reported that “patients are invited to express opinions”. The data suggest that there were no systematic differences in the way hospitals implemented patient-centredness strategies among wards, since none of the differences observed at the ward level was statistically significant.

Table 4 presents the results of logistic regression analysis to estimate the implementation of patient-centredness strategies at the ward level by maturity classification, hospital type, hospital ownership and ward type. The last column shows the association of patient-centredness strategies by maturity classification, adjusted for the remaining hospital characteristics and country. The odds ratios (ORs) reported in the table reflect implementation deficiencies, which means that higher ORs suggest that a given strategy was less likely to be implemented.

**Table 4:** Crude and adjusted ward-specific implementation of patient-centredness strategies

Domain Item	Crude maturity classification		Adjusted* maturity classification	
	High = 1 Intermediate: OR (95% CI)Low: OR (95% CI)	p Value†	High = 1 Intermediate: OR (95% CI)Low: OR (95% CI)	p Value†
Patients’ rights				
Patients give written consent to treatment	1	0.012	1	0.645
	1.6 (1.1 to 2.3)		1.3 (0.8 to 2.2)	
	2.1 (1.2 to 3.4)		1.2 (0.6 to 2.6)	
Consultation and treatment allow privacy	1	0.231	1	0.175
	1.3 (0.9 to 2.0)		1.5 (1.0 to 2.4)	
	1.5 (0.9 to 2.5)		1.5 (0.8 to 2.7)	
Patient information and empowerment				
Ability to inform foreign patients about their condition and treatment	1	0.001	1	0.030
	1.9 (1.2 to 2.9)		1.7 (1.1 to 2.8)	
	2.6 (1.6 to 4.4)		2.2 (1.2 to 3.8)	
Patients informed at discharge about follow-up care	1	0.434	1	0.202
	1.5 (0.7 to 3.2)		1.3 (0.5 to 3.1)	
	1.0 (0.4 to 2.3)		0.5 (0.2 to 1.5)	
Learning from patients				
Patients invited to express opinion	1	0.548	1	0.056
	1.1 (0.7 to 1.3)		1.3 (0.8 to 2.1)	
	1.3 (0.8 to 2.0)		2.1 (1.1 to 4.0)	
Patients informed about the complaints procedure	1	<0.001	1	<0.001
	1.8 (1.2 to 2.7)		1.8 (1.1 to 2.7)	
	4.6 (2.7 to 7.8)		3.7 (2.0 to 7.0)	
Patient hotel services				
Single room upon request	1	0.001	1	<0.001
	1.1 (0.8 to 1.6)		1.1 (0.7 to 3.8)	
	2.3 (1.4 to 3.6)		3.2 (1.7 to 3.1)	
Choice in timing of meals	1	0.006	1	0.060
	3.2 (1.2 to 8.4)		2.6 (1.0 to 7.1)	
	5.1 (1.9 to 13.9)		3.7 (1.3 to 10.8)	

*Adjusted for: country (Belgium, Czech Republic, France, Ireland, Poland, Spain), hospital type (university hospital, general teaching hospital, general non-teaching hospital), hospital ownership (public, private not-for-profit, private for-profit), and ward (acute myocardial infarction, appendicitis).

†p Value for Wald statistic.

OR, odds ratio.

For the maturity classification we observed a general negative association of implementation with intermediate or low maturity, with hospitals in the high maturity classification used as the reference group. For example, hospitals with intermediate and low maturity classification were, respectively, 1.6 (95% CI 1.1 to 2.3) and 2.1 (95% CI 1.2 to 3.4) times less likely to ensure patients’ written consent for treatment. The results for hospital type were more ambiguous. For example, university hospitals appeared less likely to provide comprehensive information about the patient’s condition and treatment than general teaching (OR 0.5; 95% CI 0.3 to 0.7) and non-teaching hospitals (OR 0.5; 95% CI 0.3 to 0.8) (data not reported in the table). On the other hand, general teaching and non-teaching hospitals were less likely to provide single rooms on request (OR 1.5; 95% CI 1.0 to 2.3 and OR 1.9; 95% CI 1.2 to 3.1, respectively). We observed statistically significant differences for patient-centredness strategies and type of hospital ownership for five of the eight items. With regard to wards, we observed significant differences only for one item (written consent to treatment); in general, there appeared to be no association with maturity classification depending on whether the ward was for AMI or appendicitis patients.

Given that some of the associations observed for type of hospital, hospital ownership, ward, and country might have confounded the association between implementation of patient-centredness strategies and the revised maturity classification, we entered these variables into a logistic regression model as controls (table 4). We observed that some of the crude associations observed for patient-centredness strategies and maturity classification disappeared after entering hospital characteristics and country into the model. Moreover, adjustment appeared to reduce the strength of association for the remaining items. Nevertheless, for “informing patients about their condition and treatment”, “informing patients about the complaints procedure”, and “providing single rooms on request”, we continued to observe a significant positive association with the revised maturity classification.

## DISCUSSION

In a large sample of European hospitals, we investigated the implementation of patient-centredness and associated characteristics of hospital structure and quality management systems. In general, hospitals in the highest maturity classification group reported better implementation of strategies to improve patient-centredness, and we found significant differences for 13 out of 18 dependent variables (patient-centredness strategies) and maturity classification at the hospital level.

The absolute level of self-reported implementation differed for each of the items examined. For example, irrespective of the classification of quality improvement maturity, hospitals reported high implementation of patients’ rights, but low involvement of patients in quality management issues. We also noted systematic differences in “learning from patients” through the use of surveys on patients’ views, despite the widespread use of such instruments.^27^ Hospitals in the highly developed maturity group appeared to make better use of the knowledge generated for organisational learning and continuous improvement. The strength of association between implementation of patient-centredness strategies and maturity, however, appeared to be lower at the ward level than at the hospital level. Moreover, some of the associations detected disappeared or were weaker after adjustment for potential confounding variables.

In our final model, we detected associations between maturity classification and patient-centredness with regard to “ability to inform patients about treatment”, “information to patients about the complaints procedure”, and “providing a single room upon request”. This suggests that the association between patient-centredness and maturity classification was confounded by country-specific quality strategies. For example, hospitals from countries with an obligatory national hospital accreditation system might have reported better implementation of some patient-centredness strategies, because these centres are also subject to national accreditation processes.^28^ On the other hand, it should be considered that the association between maturity classification and patient-centredness strategies seemed to be much stronger at the hospital level than at the ward level. In other words, hospitals might implement policies at a strategic level to meet legislative and accreditation requirements; however, the implementation of such polices might not be as strong at the ward level.^29^ The results of our study thus suggest that, although quality improvement systems appear to be effective with regard to the implementation of selected patient-centredness strategies, they are not sufficient to ensure widespread implementation of patient-centredness throughout the organisation.

Some of the findings of our study are difficult to contextualise given the lack of comparable research and the difficulty of conceptualising patient-centredness.^30^ Previous studies have identified differences in health system responsiveness,^19^ ^31^ ^32^ and a substantial literature supports patients’ concerns about the lack of patient-centredness of hospital services.^33^^–^35 However, a comparative analysis of the impact of different quality mechanisms on patient-centredness strategies (or quality strategies as a whole) at the hospital and ward level is an under-researched topic.^36^ ^37^

There are a number of limitations of the study that merit discussion. First, despite the many attempts to recruit hospitals randomly, this proved unfeasible in some countries. Moreover, the number of hospitals recruited was not sufficient to make generalisable statements about the country-specific implementation of strategies to improve patient-centredness. However, in view of the regionalisation of health systems, and of common experiences with the misclassification of hospitals within countries, it was not the purpose of this study to generalise findings at the country level.

A second limitation of the study is the incomplete operationalisation of the concept of patient-centredness. For example, in our study we were not able to include dimensions of patient-centredness such as “exploring the patients’ main concern” and “finding common ground and mutually agreeing on the management of the condition”, as suggested by Steward *et al*,^11^ or issues such as “emotional support” and “alleviating fear and anxiety”, included in the Picker approach to assessing patient-centred care.^12^ The main reason for excluding these dimensions of patient-centredness was that in this project we did not collect data at the patient level. Although these concepts have been substantially examined in the literature on shared decision making^38^ and assessing patients’ views or experiences,^27^ the link to quality improvement systems so far has not been studied in depth.

A third limitation of our study is that it is based on self-reported data and thus prone to the biases associated with self-assessment. By comparing responses to the questionnaire and subsequent audit data, we have demonstrated reasonable agreement (MARQuIS project consortium, unpublished results, Barcelona, 2006).

A final limitation is the lack of outcome measures on the actual views of patient regarding patient-centredness at hospitals that participated in the study. In light of the undeniable importance of including the patients’ views in a study of patient-centredness, we draw readers’ attention to recent calls in the literature to address the “forgotten” structural dimension in hospital quality management.^39^ ^40^ Despite these shortcomings, we believe that the provisional results presented in this article are an important contribution to the debate on the evaluation of quality improvement systems, and to the particular focus on improving patient-centredness.

From a policy perspective, the widespread implementation of policies to ensure patients’ rights, privacy, and confidentiality is noteworthy. Patient involvement in quality improvement activities, on the other hand, so far appears to be a more rhetorical exercise than a practice.^41^ Moreover, the contextual meaning of patient involvement in quality improvement activities in different hospitals and different countries needs to be explored further. A tentative finding of the study is that more mature quality improvement systems appear to be positively associated with the implementation of several strategies to improve patient-centredness. The findings are weak in the sense that they appear to be confounded by certain hospital characteristics and national quality strategies, and associations are more likely to be detected at the strategic level, whereas the strength of association is reduced at the ward level. Moreover, the association appears to be significant only for selected items of patient-centredness, and the question remains of how to ensure the implementation of a broad vision of patient-centred care.^42^ From a research perspective, it should be emphasised that the link between strategies to improve patient-centredness and the maturity of the hospital quality improvement classification is not yet well understood, and that substantial further research is required to understand these links. Further research should address the development of a score of patient-centredness strategies, and conduct comparisons of hospitals that implement strategies successfully across wards and those that adopt strategies only at the hospital policy level.

## CONCLUSION

This article presents the findings of a study of the implementation of strategies to improve patient-centredness in European hospitals, and the association between these strategies and the development of a hospital’s quality improvement system. On the one hand, some patient-centredness strategies appear to be widely implemented, such as patients’ rights and privacy; on the other hand, strategies on patients’ involvement in quality improvement activities, and strategies to learn from patients, are not widely implemented. Although several patient-centredness strategies appear to be positively associated with the development of hospital quality improvement systems (such as providing information on the patient’s condition and treatment, information on complaints procedures and single rooms on request), other strategies appear to be confounded by hospital characteristics and different national or regional quality strategies.
